# The SlHYL1‐SlRBP1 module integrates gibberellin metabolism to promote plant height in tomato (*Solanum lycopersicum*)

**DOI:** 10.1111/tpj.71210

**Published:** 2026-11-01

**Authors:** Xingxing Zhang, Miao Chai, Linjing Cao, Shengbo Chen, Kun Zhao, Jiazhen Han, Ning Li, Hongjiao Zhao, Shengjun Feng, Bo Wang, Shengze Zhang, Yumeng Li, Mingze Xu, Guodong Ren, Shuai Gao

**Affiliations:** ^1^ Key Laboratory of Quality and Safety Control for Subtropical Fruit and Vegetable, Ministry of Agriculture and Rural Affairs, College of Horticulture Zhejiang A&F University Hangzhou 311300 China; ^2^ College of Life Sciences Zaozhuang University Zaozhuang 277160 China; ^3^ College of Advanced Agricultural Sciences Zhejiang Wanli University Ningbo 315100 China; ^4^ State Key Laboratory of Genetics and Development of Complex Phenotypes, Institute of Plant Biology, School of Life Sciences Fudan University Shanghai 200438 China

**Keywords:** SlHYL1, SlRBP1, plant height, GA, miRNA biogenesis, tomato

## Abstract

Plant height is an essential agronomic trait that significantly influences crop yield and quality. The HYPONASTIC LEAVES 1 (HYL1), a core component of microRNA biogenesis, remains poorly understood in terms of its regulatory mechanisms on agronomic traits in tomato (*Solanum lycopersicum*, Sl). In this study, we demonstrated that CRISPR/Cas9‐mediated knockout of *SlHYL1* caused dwarfism, and partial suppression of *SlHYL1* in tomato cultivar “Ailsa Craig” (AC) via RNA interference (RNAi) increased internode number but reduced internode length, resulting in a semi‐dwarf phenotype, confirming the role of SlHYL1 in regulating plant height. Cytological analysis showed that *SlHYL1* suppression decreased both cell number and size within internodes. Yeast two‐hybrid screening identified that SlHYL1 interacted with another tomato RNA‐binding protein 1 (SlRBP1). Knockdown of *SlRBP1* resembled the *SlHYL1‐RNAi* semi‐dwarf phenotype. Further studies revealed that the SlHYL1‐SlRBP1 module additively promotes the expression of gibberellin (GA) biosynthetic genes, thereby facilitating GA accumulation. Additionally, the dwarf phenotypes of both *SlHYL1‐RNAi* and *SlRBP1‐RNAi* plants were partially rescued by exogenous GA_3_ application. Notably, unlike SlHYL1, SlRBP1 did not affect the biogenesis of most miRNAs. In summary, our findings demonstrate that the SlHYL1‐SlRBP1 module regulates tomato plant height by enhancing GA accumulation.

## Significance Statement

Plant height is a crucial agronomic trait; however, the molecular mechanism by which dsRBP regulates tomato plant height remains unclear. This study demonstrates that SlHYL1 and its interacting protein SlRBP1 positively regulate the expression of GA‐biosynthetic genes *SlCPS* and *SlKAO* through additive effects, thereby promoting tomato plant height. The research elucidates the molecular mechanism of the SlHYL1‐SlRBP1 module in regulating tomato plant height, providing new theoretical insights for crop architecture regulation.

## INTRODUCTION

Plant height is a key architectural and agronomic trait that directly affects harvest index and yield potential (Yang & Hwa, 2008; Zhou, Li, et al., 2023). The introduction of dwarfing traits during the “Green Revolution” significantly increased wheat and rice yields. Additionally, in protected cultivation, height regulation by topping improves light penetration, ventilation, and management, further enhancing yield. These examples highlight the critical role of plant height in increasing agricultural production efficiency (Hedden, 2003; Peng et al., 1999).

Plant height is influenced by both environmental factors and phytohormones, with gibberellins (GAs) playing a crucial role (Davière et al., 2014; Hauvermale et al., 2022). To date, only a few types of GAs (GA_1_, GA_3_, GA_4_, and GA_7_) have demonstrated biological activity in plants (Gong et al., 2025). GA biosynthesis, catabolism, and signal transduction have been extensively studied (Wang et al., 2017). GA biosynthesis begins with geranyl geranyl diphosphate (GGDP), which is transformed into ent‐kaurene by ent‐copalyl diphosphate synthase (CPS) and ent‐kaurene synthase (KS), and then into GA_12_ via ent‐kaurene oxidase (KO) and ent‐kaurenoic acid oxidase (KAO) (Yamaguchi, 2008). From this point, two parallel pathways emerge: one convers GA_12_ to GA_53_ by GA13‐oxidase (GA13ox), then to GA_1_ and GA_3_ via GA 20‐oxidase (GA20ox) and GA3‐oxidase (GA3ox); the other converts GA_12_ directly to GA_4_ by GA20ox and GA3ox. GA inactivation is mediated by GA2‐oxidases (GA2oxs), which degrade either 19‐carbon GAs (e.g., GA_1_, GA_4_) or 20‐carbon GAs (e.g., GA_12_, GA_53_) (He et al., 2020; Hedden & Thomas, 2012; Serrani et al., 2007). Endogenous GA levels are tightly controlled by the coordinated actions of biosynthesis and catabolism. Generally, the lower the GA level, the higher the degree of plant dwarfing (Shani et al., 2024). Regarding signal transduction, loss‐of‐function mutations often cause GA‐insensitive dwarfism, whereas gain‐of‐function mutations lead to slender phenotypes (Sponsel, 2018).

MicroRNAs (miRNAs), which are a class of non‐coding RNA, typically 21–24 nucleotides in length, that play essential roles in plant growth, development, and response to abiotic and biotic stresses (Li et al., 2017; Zhang, Xiang, et al., 2022). HYL1 is a core component of the miRNA processing complex, where it functions alongside Dicer like 1 (DCL1) and Serrate (SE) to promote accurate and efficient miRNA biogenesis (Dong et al., 2008; Han et al., 2004). Many miRNAs have been implicated in plant height regulation. For example, miR156/157 regulates vegetative phase transition (Wu et al., 2009), miR166 modulates GA metabolism by targeting homeodomain transcription factors in soybean (Zhao et al., 2022), and miR319 overexpression reduces plant height in rice (Liu et al., 2017). Despite their significant influence, many miRNAs in horticultural crops remain uncharacterized, and the underlying regulatory mechanisms are largely unknown. In this research, we investigated the role of SlHYL1 and its interacting partner SlRBP1 in tomato plant height regulation. We provide evidence that the SlHYL1‐SlRBP1 module modulates GA accumulation to promote stem elongation, and that SlRBP1 functions largely independently of the canonical miRNA biogenesis pathway in this context.

## RESULTS

### *SlHYL1* is ubiquitously expressed in various tissues and responds to multiple exogenous hormones

The tomato genome contains eight *DRB* (*Double‐Stranded RNA‐Binding*) genes (Huang et al., 2022). Gene expression analysis revealed that six *SlDRBs* (*DRB1, 2, 3, 4, 7*, and *8*) are robustly expressed in all organs examined—roots, leaves, flowers, and fruits—according to data from the Tomato Expression Database (https://ted.bti.cornell.edu/) (Figure S1a). *SIDRB5* is only weakly expressed in leaves and flowers, whereas *SIDRB6* shows no detectable expression in all tissues, indicative of a pseudogene. Among these expressed *SlDRB* genes, *SlDRB1* has drawn our attention for two reasons. Firstly, *SlDRB1* exhibited relatively high expression levels across multiple organs. Secondly, SlDRB1 protein lacks the C‐terminal tandem repeats compared to AtDRB1 in *Arabidopsis*, suggesting that SlDRB1 may possess distinct functions (Figure S1b). So, we focused on *SlDRB1* (*Solyc04G076420*) and designating it as *SlHYL1* due to its closest phylogenetic relationship with *Arabidopsis* HYL1, and its counterparts in *Chinese cabbage* and Apple (Figure S1c) (Ren et al., 2020; Shen et al., 2022). To obtain a more detailed expression profile of *SlHYL1*, we utilized qRT‐PCR to quantify its transcript levels in seven tissues: roots, stems, shoot apical meristem, mature leaves, young leaves, flowers, and fruits. *SlHYL1* is ubiquitously expressed, with highest expression in flowers and fruits (Figure S2a). Exogenous GA_3_ treatment transiently upregulated *SlHYL1*, peaking at 8 h post treatment and declined thereafter. By contrast, IAA and KT treatment only slightly downregulated *SlHYL1* expression (Figure S2c–e).

Furthermore, we investigated the subcellular localization of SlHYL1 by transient expression in tobacco leaves. Similar to the observation in *Arabidopsis* (Fang & Spector, 2007; Zhang et al., 2017), the GFP‐SlHYL1 fusion protein was detected both in the nucleus and cytoplasm, suggesting that SlHYL1 may also be a nucleocytoplasmic shuttling protein (Figure S2b).

### Partial suppression of *SIHYL1* results in semi‐dwarf plant height

To elucidate the native function of SlHYL1 in tomato, we first generated knockout mutants of *SlHYL*1 using CRISPR‐Cas9‐induced gene editing. Three types of homozygous mutants were isolated and designated as *Slhyl1‐1*, *Slhyl1‐2*, and *Slhyl1‐3* (Figure S3a). All three mutants exhibited varying degrees of gene fragment deletions, specifically a 4 bp deletion for *Slhyl1‐1*, an 80 bp deletion for *Slhyl1‐2*, and a 1464 bp deletion for *Slhyl1‐3*. These deletions resulted in frameshifts within the *SlHYL1* coding region, leading to premature translation termination of the SlHYL1 protein (Figure S3b). Notably, all three *Slhyl1* mutants displayed a dwarf phenotype, and the severe loss of SlHYL1 function also resulted in the loss of leaf polarity, culminating in long narrow leaves devoid of lobules (Figure S3c). Statistical analysis revealed that the average plant height of these *SlHYL1* knockout plants was approximately 5 cm, compared to 18.5 cm for the control AC plants (Figure S3d). Together, these findings suggest that the dysfunction of SlHYL1 leads to developmental abnormalities in tomato.

Due to the severe developmental defects of the mutants and their inability to produce progeny, we developed knockdown transgenic lines (*SlHYL1‐RNAi*) via RNA interference in the AC background. Additionally, we constructed *SlHYL1* overexpression (*SlHYL1‐OX*) transgenic lines. qRT‐PCR analysis confirmed efficient knockdown and overexpression, respectively (Figure S4a,b). Western blot assay further confirmed the expression of GFP‐SIHYL1 fusion protein in *SlHYL1‐OX* plants (Figure S4c). For subsequent experiments, *SlHYL1‐RNAi* lines 17# and 27# and *SlHYL1‐OX* lines 2# and 3# were selected. *SlHYL1‐RNAi* lines were markedly dwarf compared to AC controls, whereas *SlHYL1‐OX* plants displayed no obvious morphological alterations (Figure 1a,b). The height of the plant is influenced by the length and quantity of its internodes; therefore, we recorded the lengths of the first five internodes and tallied the total number of internodes for each genotype. The results showed that *SlHYL1‐RNAi* lines exhibited significantly shorter internodes but an increase in internode number, whereas *SlHYL1‐OX* plants did not affect the height (Figure 1c–e).

**Figure 1:**
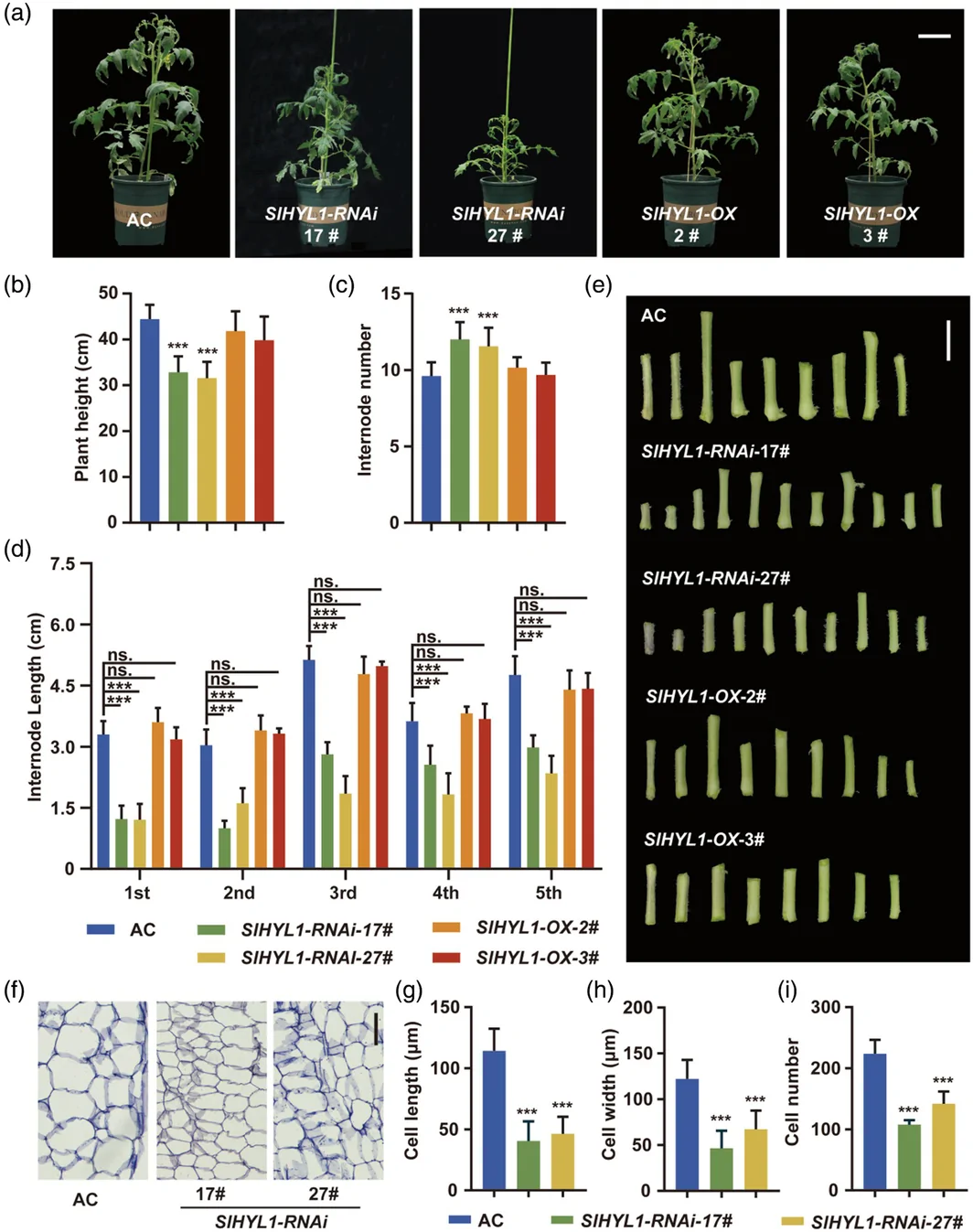
Phenotypes of *SlHYL1* transgenic lines in tomato. (a, e) 6‐week‐old AC and *SlHYL1* transgenic lines of seedling (a) and internode (e). Scale bar = 10 cm in A, 2 cm in e. (b–d) Plant height (b), internode number (c) and length of first five internodes (d). In (b–d), each value represents the means ± SD (*n* > 15). Asterisks indicate statistically significant differences between transgenic lines and AC by *t*‐test (***P < 0.001). (f) Longitudinal section of the AC and two *SlHYL1* interference plants. Scale bar = 100 μm. (g–i) Cell length (g), cell width (h), and cell number (i) of the second internode in AC and two *SlHYL1* interference plants. In (g–i), each value represents the means ± SD (*n* > 100). Asterisks indicate statistically significant differences between transgenic lines and AC by *t*‐test (ns = no significance; ***P < 0.001).

To determine whether the height reduction resulted from defective cell elongation and/or proliferation, we investigated the size as well as number of cells in longitudinal sections from the second internode of both *SlHYL1‐RNAi* lines and the control AC utilizing a paraffin‐section assay. The results showed that knockdown of *SlHYL1* decreased both cell size (length and width) and cell number. Mean cell length in stems of *SlHYL1‐RNAi‐17#* and *SlHYL1‐RNAi‐27#* stem was 35.4% and 40.62% of that in AC, respectively; width was 38.05% and 54.98%; and cell number was 48.10% and 63.53% of the control values (Figure 1f–i). Thus, knockdown of *SlHYL1* reduces plant height by impairing both cell elongation and proliferation.

### SlHYL1 physically interacts with SlRBP1

To investigate the molecular mechanism underlying SlHYL1‐mediated regulation of plant height in tomato, we performed yeast two‐hybrid (Y2H) screening of a tomato cDNA library using SlHYL1 as a bait. Among the candidate proteins (Table S1), SlRBP1 (Solyc10g109660), annotated as glycine‐rich RNA‐binding protein 1, was selected for further study because dysfunction of SlRBP1 also reduces plant height (Ma et al., 2022).

To validate the interaction and determine which region of SlHYL1 is required for interaction, we reperformed Y2H assays with full‐length SlHYL1 (SlHYL1) and three truncated variants (SlHYL1^D1^, SlHYL1^D1D2^, and SlHYL1^C^) fused to the GAL4 DNA‐binding domain (BD‐SlHYL1, BD‐SlHYL1^D1^, BD‐SlHYL1^D1D2^, and BD‐SlHYL1^C^), along with SlRBP1 fused to the activation domain (AD‐SlRBP1). The results showed that both full‐length SlHYL1 and the SlHYL1^C^ variant interacted with SlRBP1 (Figure 2a).

**Figure 2:**
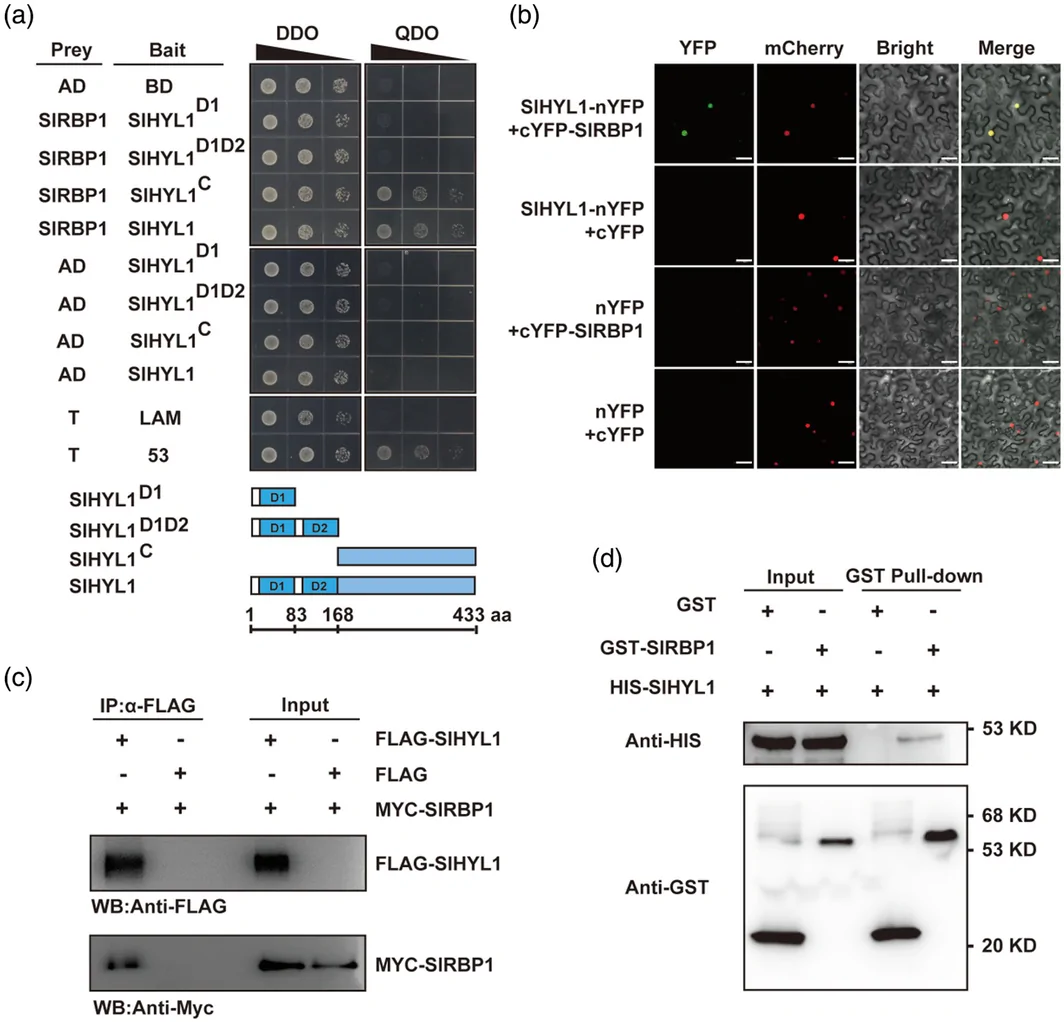
SlHYL1 physically interacts with SlRBP1. (a) Y2H assay of the interaction between SlRBP1 and SlHYL1 in yeast. (b) BiFC assay reveal that SlHYL1 interacts with SlRBP1 in *Nicotiana benthamiana* leaves. NLS‐mCherry indicates nuclear localization. Bar = 50 μm. (c) Co‐IP assay of the interaction between SlHYL1 and SlRBP1. FLAG‐tagged SlHYL1 (FLAG‐SlHYL1) and MYC‐tagged SlRBP1 (MYC‐SlRBP1) or a FLAG empty control were transiently co‐expressed in *Nicotiana benthamiana* leaves. The extracts were immunoprecipitated using anti‐FLAG beads, and the proteins were detected by immunoblotting with anti‐FLAG and anti‐MYC antibodies. (d) GST pull‐down assay of the interaction between SlHYL1 and SlRBP1 in vitro. HIS fused SlHYL1 (HIS‐SlHYL1) was tested for binding to GST fused SlRBP1 (GST‐SIRBP1), with GST alone serving as the negative control.

We further verified the interaction using bimolecular fluorescence complementation (BiFC) in tobacco leaves, pull‐down, and co‐immunoprecipitation (Co‐IP) assays. In the BiFC assay, reconstituted YFP signals were exclusively detected in the nucleus when SlHYL1‐nYFP and cYFP‐SlRBP1 were co‐infiltrated, suggesting that the interaction takes place within the nucleus (Figure 2b). Co‐IP analysis by transiently expressing FLAG‐tagged SlHYL1 (SlHYL1‐FLAG) and MYC‐tagged SlRBP1 (MYC‐SlRBP1) in tobacco leaves further validated the interaction (Figure 2c). Furthermore, in the pull‐down assay, GST‐SlRBP1, but not GST alone, captured HIS‐SlHYL1 (Figure 2d). Collectively, these findings demonstrate that SlHYL1 directly interacts with SlRBP1 both *in vitro* and *in vivo*.

### *SlRBP1* knockdown phenocopies *SlHYL1‐RNAi* lines

Based on the above findings, we hypothesized that SlRBP1 may act as a cofactor of SlHYL1 in regulating plant height. To test this hypothesis, we knocked down *SlRBP1* in AC using RNAi. qRT‐PCR confirmed efficient silencing in five independent transgenic lines (*SlRBP1‐RNAi‐8#*, *11#*, *12#, 21#*, and *22#*), showing a reduction in *SlRBP1* expression levels of 58.90%, 67.90%, 46.57%, 56.10%, and 36.10% relative to the AC level, respectively (Figure S5).

Lines *SlRBP1‐RNAi‐8#*, and ‐*11#*, which showed the strongest suppression of *SlRBP1* expression, were selected for phenotypic analyses. As shown in Figure 3a,b, *SlRBP1‐RNAi‐8#* and ‐*11#* plants exhibited significantly reduced plant height. Specifically, the heights of *SlRBP1‐RNAi‐8#* and ‐*11#* plants were 24.68 cm and 20.4 cm, corresponding to 55.59% and 45.95% of the control height, respectively (Figure 3a,b).

**Figure 3:**
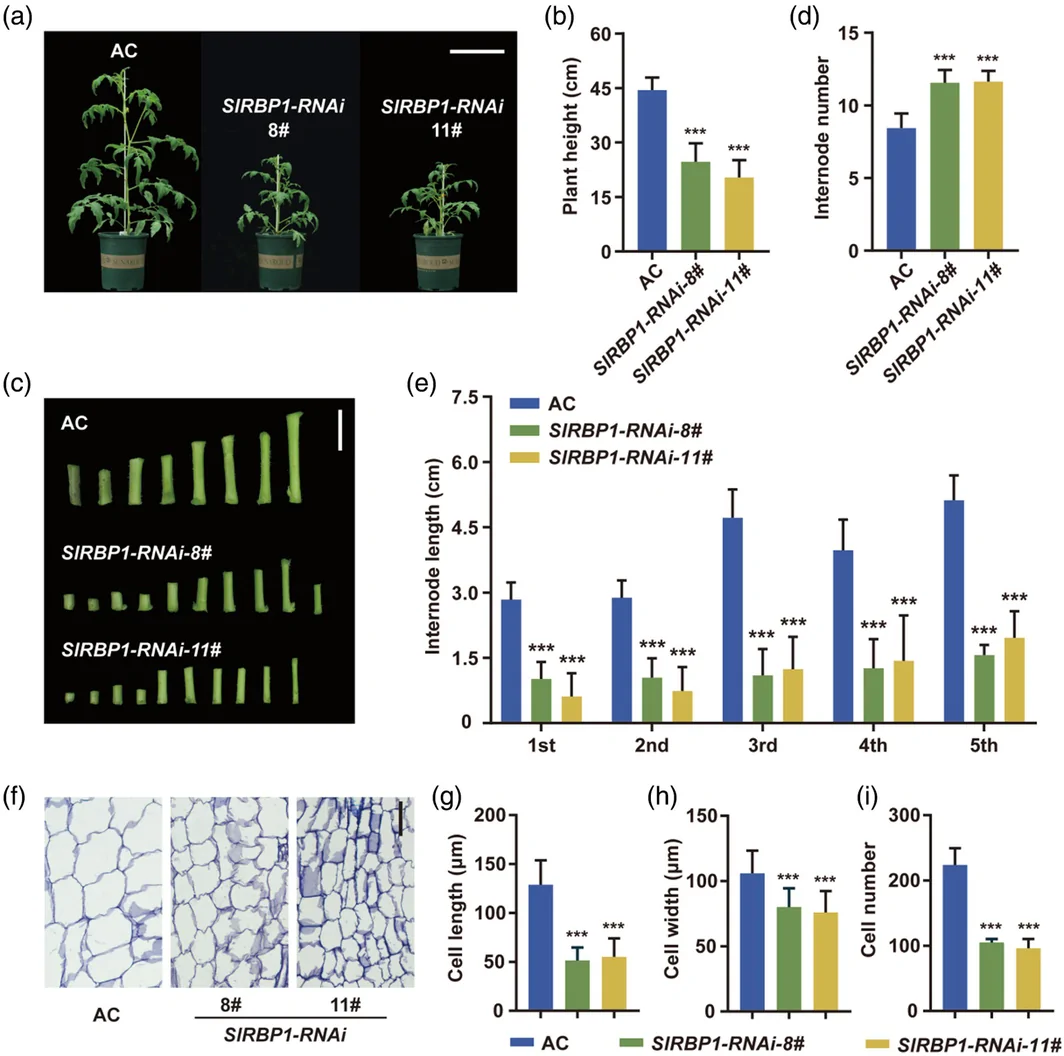
Phenotypes of *SlRBP1* interference lines in tomato. (a, c) 6‐week‐old *SlRBP1* interference lines (*SlRBP1‐RNAi‐8#*, *SlRBP1‐RNAi‐11#*), wild type (AC) of seedling (a) and internode (c). Scale bar = 10 cm in A, 3 cm in C. (b, d, e) Plant height (b), internode number (d), and length of first five internodes (e). In (b, d, e), each value represents the means ± SD (*n* = 15). Asterisks indicate statistically significant differences between transgenic lines and AC by *t*‐test (***P < 0.001). (f) Longitudinal section of the AC, and two *SlRBP1* interference lines plants. Scale bar = 100 μm. (g–i) Cell length (g), cell width (h), cell number (i) of the second internode in AC and two *SlRBP1* interference lines. In (g–i), each value represents the means ± SD (*n* > 100. Asterisks indicate statistically significant differences between transgenic lines and AC by *t*‐test (*P < 0.05, **P < 0.01, ***P < 0.001).

We then counted internodes and measured the first five internodes' length in each genotype. Similar to *SlHYL1‐RNAi* plants, *SlRBP1‐RNAi* plants had more internodes, but each internode was shorter (Figure 3c–e). Inspection of longitudinal paraffin sections of the second internode revealed that *SlRBP1‐RNAi* plants reduced both cell size and cell number (Figure 3f–i). We also examined whether SlHYL1 and SlRBP1 regulate each other at the transcriptional level. qRT‐PCR analysis showed that the expression levels of *SlHYL1* and *SlRBP1* in each other's transgenic lines did not differ significantly from those observed in the WT (Figure S6a,b). Together, these results indicate that SlHYL1 and SlRBP1 do not transcriptionally regulate each other, supporting that their functional cooperation is likely mediated by protein–protein interaction rather than transcriptional regulation.

### Transcriptome comparison between *SlHYL1‐* and *SlRBP1‐* RNAi lines

To test whether SlHYL1 and SlRBP1 act in the same pathway to regulate plant development, we performed RNA sequencing on the shoot apical meristem of two‐week‐old AC, *SlHYL1‐RNAi‐17#*, and *SlRBP1‐RNAi* ‐8# plants. Using thresholds of an absolute fold change >1.5 and *q*‐value <0.05, we identified 2312 differentially expressed genes (DEGs) in *SlHYL1‐RNAi* and 2250 in *SlRBP1‐RNAi* relative to AC (designated as SlHYL1‐ and SlRBP1‐regulated genes, respectively) (Table S2). Among these, 1673 were upregulated and 639 downregulated in *SlHYL1‐RNAi*, while 1267 were upregulated and 983 downregulated in *SlRBP1‐RNAi* (Figure S7a,b; Table S2). We next compared the transcriptome changes between *SlHYL1‐RNAi* and *SlRBP1‐RNAi* through the use of t‐distributed stochastic neighborhood embedding (t‐SNE) tool (Zhang, Wu, et al., 2022). The analysis revealed that *SlHYL1‐RNAi* and *SlRBP1‐RNAi* samples were clustered closely together but were distantly separated from AC along both dimensions (Figure S7c), suggesting that knockdown of *SlHYL1* and *SlRBP1* results in highly similar transcriptome changes. A Venn diagram analysis uncovered 1253 co‐regulated genes, accounting for 54.2% of SlHYL1‐regulated and 55.7% of SlRBP1‐regulated genes (*P* value = 0) (Figure 4a). Among these, 891 genes were co‐upregulated and 341 genes co‐downregulated. By contrast, only 13 genes were upregulated in *SlHYL1‐RNAi* but downregulated in *SlRBP1‐RNAi*, and none was downregulated in *SlHYL1‐RNAi* but upregulated in *SlRBP1‐RNAi* (Figure 4a). Thus, 98.96% (1240 of 1253) of co‐regulated genes were modulated in the same direction by SlHYL1 and SlRBP1. Hierarchical clustering of the 1253 co‐regulated genes further confirmed the concordant regulatory effect by SlHYL1 and SlRBP1 (Figure 4b). These highly similar transcriptome changes between *SlHYL1‐RNAi* and *SlRBP1‐RNAi* strongly suggest that SlHYL1 and SlRBP1 act in the same pathway.

**Figure 4:**
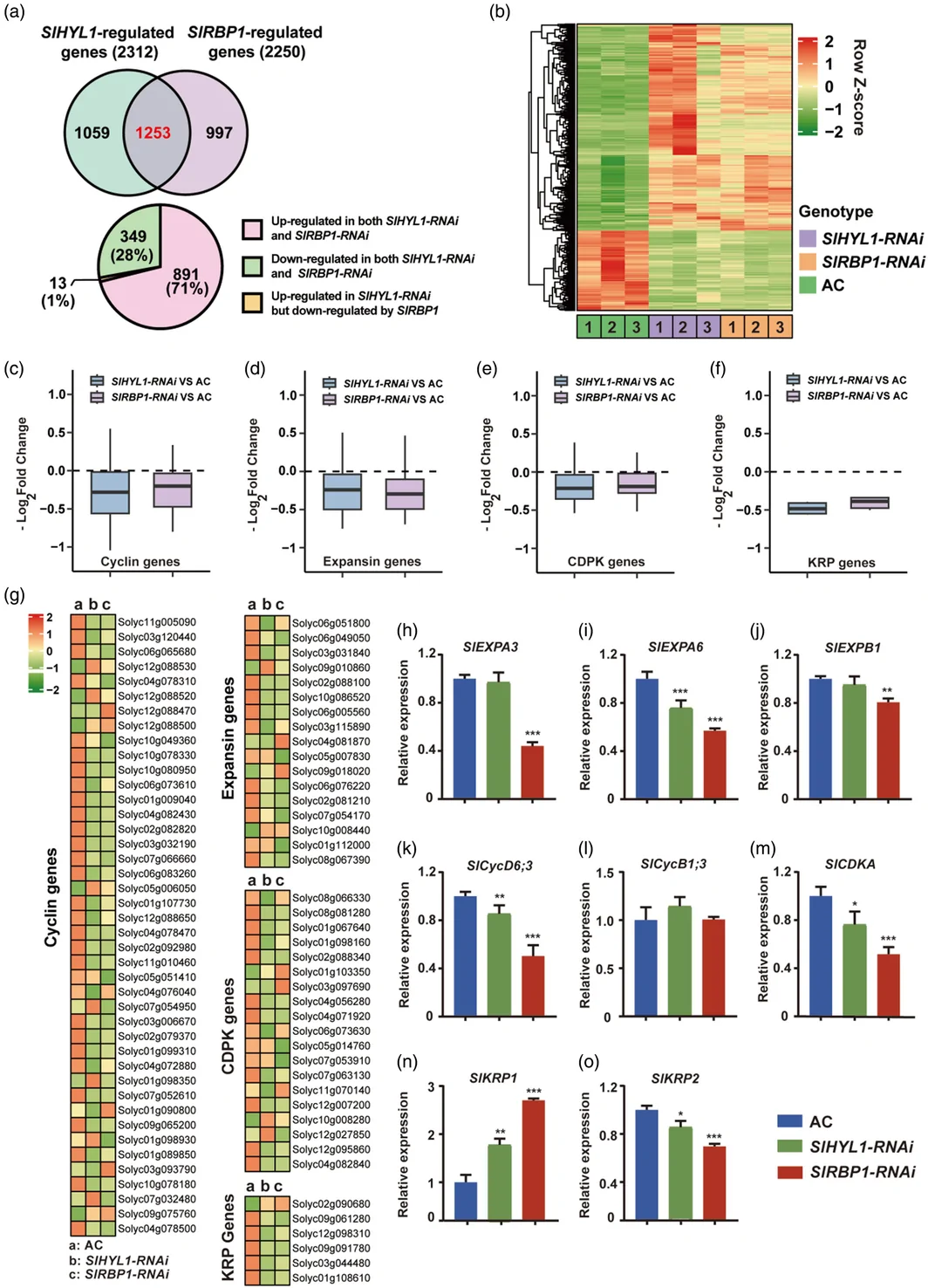
Transcriptome and qRT‐PCR analysis of SlHYL1 and SlRBP1 co‐regulated gene. (a) The Venn diagram showing the SlHYL1‐ and SlRBP1‐regulated genes and the distribution of genes upregulated and/or downregulated by SlHYL1 and SlRBP1. Genes in the overlapping region were identified as co‐regulated genes of SlHYL1 and SlRBP1. (b) Heatmap of genes co‐regulated by SlHYL1 and SlRBP1 (*n* = 3 biological replicates). (c–f) Boxplot analysis of expansin genes and cell proliferation genes, such as Cyclin genes (c), Expansin genes (d), CDPK genes (e), and KRP genes (f). (g) Heatmap of expansin genes and cell proliferation genes regulated by SlHYL1 and SlRBP1 from RNA‐seq data. (h–o) qRT‐PCR analysis of the expression level of tomato expansin genes (h–j), Cyclin genes (k, l), Cyclin‐dependent kinase gene (m), and Kip‐related protein genes (n, o) in AC, *SlHYL1*‐*RNAi*, and *SlRBP1*‐*RNAi* lines. Data are mean ± SD, *n* = 3 biological replicates. Asterisks indicate statistically significant differences between transgenic lines and AC by *t*‐test (*P < 0.05, **P < 0.01, ***P < 0.001).

In light of the paraffin‐section results described above, we next examined the expression of genes involved in cell cycle and cell expansion, as both are essential for regulating cell size and proliferation. RNA‐seq data revealed that a significant portion of these genes, including Cyclins, Expansins, CDPKs, and KRPs, were globally downregulated in both *SlHYL1‐RNAi* and *SlRBP1‐RNAi* (Figure 4c–f). Specifically, 71.43% (30 out of 42) cyclin genes, 76.47% (13 out of 17) expansin genes, 73.68% (14 out of 19) cyclin‐dependent kinase genes, and 83.33% (5 out of 6) cyclin‐dependent kinase inhibitor genes showed reduced expression (Figure 4g). The results were validated by qRT‐PCR analysis of selected representative genes, including three expansin genes (*SlEXPA3, SlEXPA6*, and *SlEXPB1*), two cyclin genes (*SlCycD6;3* and *SlCycB1;3*), one cyclin‐dependent kinase gene (*SlCDKA*), and two cyclin‐dependent kinase inhibitor genes (*SlKRP1* and *SlKRP2*) (Figure 4h–o). Collectively, these data suggest that the SlHYL1‐SlRBP1 module promotes plant height by enhancing the transcript level of cell expansion and proliferation genes.

Given the conserved role of HYL1 in plant miRNA biogenesis, we subsequently analyzed the variation patterns of pri‐miRNAs and miRNA target genes from RNA‐sequencing data. The expression levels of 57 annotated pri‐miRNAs showed an overall upward trend in *SlHYL1‐RNAi*. Strikingly, they were largely unaffected in *SlRBP1‐RNAi* lines (Figure S7d,f). Similarly, the expression of miRNA target genes exhibited limited overall de‐repression in *SlHYL1‐RNAi* but almost none in *SlRBP1‐RNAi* lines (Figure S7e,g). These patterns were validated by qRT‐PCR analysis of ten pri‐miRNAs (*pri‐miR159a*, *pri‐miR160a*, *pri‐miR162*, *pri‐miR168a*, *pri‐miR319a*, *pri‐miR394a/b*, *pri‐miR482b/c*, and *pri‐miR9479*), and nine miRNA target genes (*Solyc06g073640*, *Solyc11g069500*, *Solyc06g072300*, *Solyc12g014140*, *Solyc08g048390*, *Solyc02g077250*, *Solyc05g015520*, *Solyc02g036270*, and *Solyc08g076000*) (Figure S7h,i). Additionally, the expression of eight mature miRNAs was detected using stem‐loop qRT‐PCR. As shown in Figure S7j, seven out of eight mature miRNAs (i.e., miR159, miR160, miR167b, miR168, miR319, miR482, and miR9479) exhibited varying degrees of reduction in *SlHYL1‐RNAi* plants, except for miR162. By contrast, *SlRBP1‐RNAi* plants displayed downregulation of only miR160 and miR9479, indicating that SlHYL1, but not SlRBP1, promotes miRNA biogenesis. Taken together, these results suggest that SlRBP1 may regulate tomato plant height independently of the SlHYL1‐mediated miRNA biogenesis pathway.

### Exogenous GA treatments rescue the semi‐dwarf phenotype of *SlHYL1* and *SlRBP1* interference lines

Given the crucial role of GAs in plant height regulation, we analyzed the transcriptional level of GAs metabolic genes from transcriptome data. Notably, two rate‐limiting GA‐biosynthetic genes, *SlCPS* and *SlKAO*, were significantly downregulated in both *SlHYL1‐RNAi* and *SlRBP1‐RNAi* (Figure 5a). We also observed altered expression of GA20 oxidase and GA3 oxidase family genes, which are essential for GA biosynthesis, as well as GA2 oxidase family genes that mediate GA catabolism (Figure 5a).

**Figure 5:**
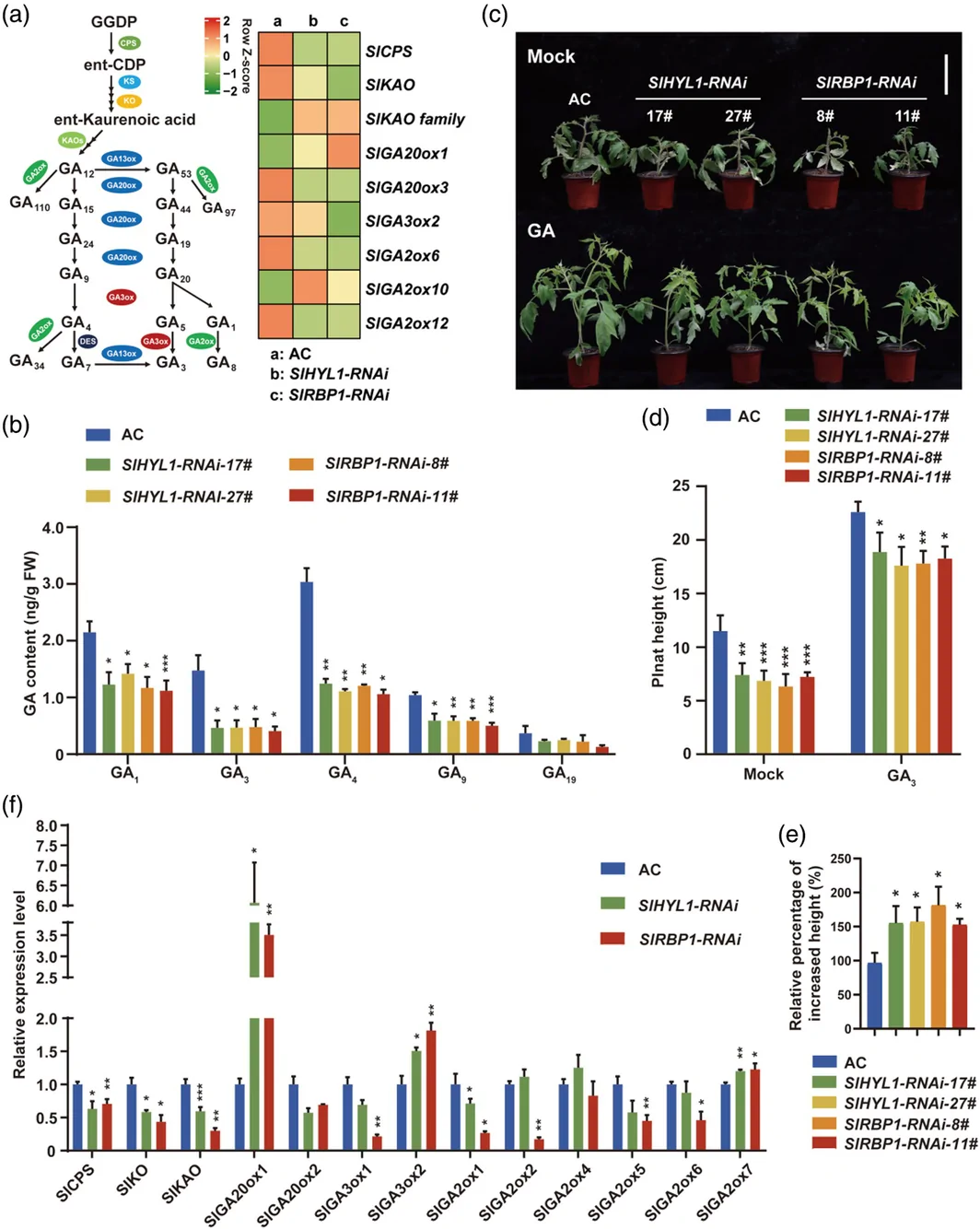
SlHYL1 and SlRBP1 regulate tomato plant height through the GA pathway. (a) Schematic diagram of GA metabolism pathways and heatmap of genes co‐regulated by SlHYL1 and SlRBP1 related to GA metabolism. (b) Content of GA compounds in AC, *SlHYL1*‐ and *SlRBP1‐* interference lines. (c) AC, *SlHYL1*‐ and *SlRBP1‐*interfering plants were treated with exogenous GA_3_, with a mock treatment serving as the control. Scale bar = 10 cm. (d) The plant height of the specimens shown in (c) is measured. (e) Percentage of increased plant height before and after GA_3_ treatment in (d). (f) Transcription level of GA‐biosynthetic and catabolic genes in AC, *SlHYL1‐* and *SlRBP1‐*interference lines. Each value represents the means ± SD (*n* > 15 in c, *n* = 3 in b, d, and f). Asterisks indicate statistically significant differences between transgenic lines and AC by *t*‐test (*P < 0.05, **P < 0.01, ***P < 0.001).

Consistent with the transcriptomic results, qRT‐PCR confirmed that the SlHYL1‐SlRBP1 module modulates the expression of key GA‐biosynthetic and GA‐catabolic genes (Figure 5f). Thus, both GA biosynthesis and degradation are perturbed in *SlHYL1*‐ and *SlRBP1‐RNAi* plants. The inconsistent expression patterns of those genes highlight the intricate GA‐metabolic network controlled by the SlHYL1‐SlRBP1 module.

To determine whether these transcriptional changes affect endogenous GA levels, we measured GAs contents in stems of *SlHYL1‐RNAi*, *SlRBP1‐RNAi*, and AC plants using liquid chromatography–mass spectroscopy/mass spectroscopy (LC–MS/MS) method. As shown in Figure 5b, the content of GA_1_, GA_3_, GA_4_, and GA_9_ in both *SlHYL1‐RNAi* and *SlRBP1‐RNAi* transgenic lines was significantly lower than that in AC (Figure 5b).

We next sprayed two‐week‐old seedlings with 100 μM GA_3_ every 48 h for 2 weeks and monitored the growth. As shown in Figure 5c–e, GA_3_ application significantly restored the height of *SlHYL1‐RNAi* and *SlRBP1‐RNAi* plants to a substantial extent. Collectively, these findings indicate that the SlHYL1‐SlRBP1 module controls plant height, at least in part, by modulating endogenous GA content.

### SlHYL1 and SlRBP1 regulate plant height in an additive manner

To further clarify the genetic connection between SlHYL1 and SlRBP1, we crossed *SlHYL1‐RNAi‐17#* with *SlRBP1‐RNAi‐8#* to obtain double‐RNAi progeny. Given that RNAi has limitations, including potential off‐target effects, we validated the expression level of *SlHYL1* and *SlRBP1* in the double‐interference lines by qRT‐PCR and found that both *SlHYL1* and *SlRBP1* were significantly downregulated in these lines (Figure 6f,g). Subsequently, we characterized the phenotype of the double‐RNAi lines. Compared to their single‐knockdown parents, the plant height of the double‐RNAi line decreased significantly (Figure 6a,b). In addition, we measured the number of internodes and the lengths of the first five internodes for each genotype. As shown in Figure 6c–e, the internode length of the double‐RNAi line was significantly shorter than that of their single‐knockdown parents, but no significant differences were observed in internode number (Figure 6c–e). These results indicate that SlHYL1 and SlRBP1 have an additive effect on promoting the height of tomato plants, primarily reflected in the length of the internodes rather than the number of internodes.

**Figure 6:**
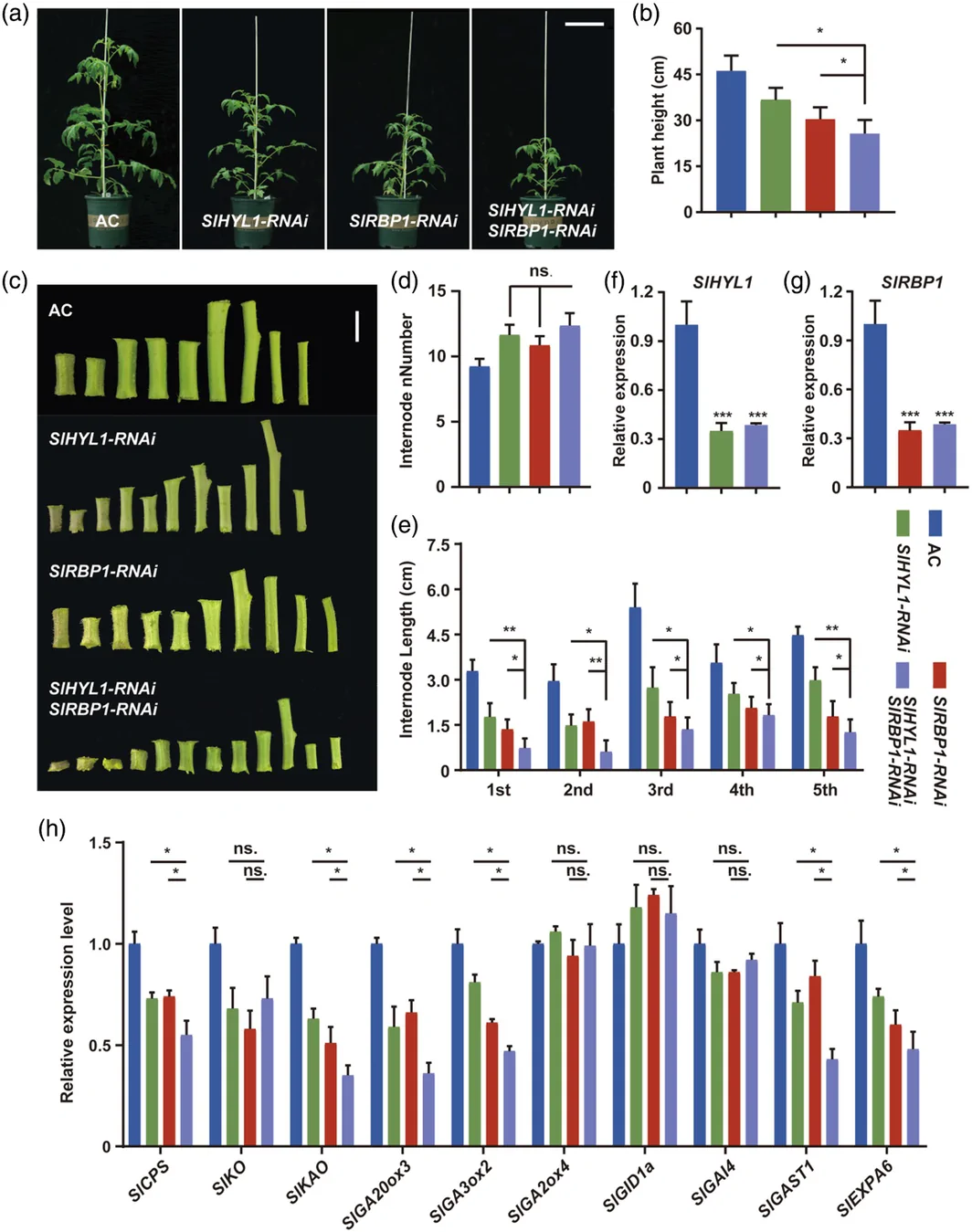
Genetic analysis of SlHYL1 and SlRBP1 in regulating plant height via GA. (a, c) 6‐week‐old AC, *SlHYL1‐RNAi, SlRBP1‐RNAi*, and double knockdown lines. Scale bar = 10 cm in (a) and 3 cm in (c). (b, d, e) Plant height (b), internode number (d), and length of first five internodes (e). (f–h) Expression level of *SlHYL1* (f), *SlRBP1* (g), and GA‐related genes (h) in different genotypes. Each value represents the means ± SD (*n* > 15 in b, d, and e; *n* = 3 in f–h). Asterisks indicate statistically significant differences between transgenic lines and AC by *t*‐test (ns = no significance, *P < 0.05, **P < 0.01, ***P < 0.001).

Furthermore, we investigated whether the simultaneous suppression of *SlHYL1* and *SlRBP1* also affects the expression of GA metabolism, signaling transduction, and response genes. The qRT‐PCR results indicated that in the double‐RNAi plants, the expression levels of two GA metabolism genes (*SlKO* and *SlGA2ox4*) and two GA‐signaling genes (*SlGID1a* and *SlGAI4*) were unchanged compared to their single‐parent counterparts, while the other four SlHYL1‐SlRBP1 co‐regulated GA metabolism genes (*SlCPS*, *SlKAO*, *SlGA20ox3*, and *SlGA3ox2*) and two GA‐responsive genes (*SlGATS1* and *SlEXPA6*) were significantly reduced compared to their single‐parent counterparts (Figure 6h). Additionally, three SlHYL1‐specific regulated genes (*SlACS2*, *SlTAGL1*, and *SlTPS3*) and three SlRBP1‐specific regulated genes (*SlCKX5*, *SlXTH3*, and *SlWRKY3*) were also detected using qRT‐PCR. Compared with the single‐parent lines, the expression levels of these genes in the double‐RNAi lines showed no significant changes (Figure S8). To further investigate the SlHYL1‐SlRBP1 module regulates GA biosynthesis directly or indirectly, we employed a dual‐luciferase transient expression system to assess the responses of the rate‐limiting enzyme genes *SlCPS* and *SlKAO* during the induction of *SlHYL1* and *SlRBP1* expression. Our findings indicated that with the increasing expression of *SlHYL1* and *SlRBP1*, the ratio of LUC/REN also exhibited an upward trend. However, the enhancement of LUC activity significantly lagged behind the expression levels of *SlHYL1* and *SlRBP1* (Figure S9), suggesting that the regulation of *SlCPS* and *SlKAO* expression by the SlHYL1‐SlRBP1 module occurs indirectly.

Moreover, to determine the function of SlHYL1 and SlRBP1 in GA synthesis or in this response, we analyze the expression of *SlHYL1* and *SlRBP1* genes in tomato GA‐deficient mutant *gib3* (Martins et al., 2025). In *gib3* mutant, the expression levels of both *SlHYL1* and *SlRBP1* were found to be decreased (Figure S10), suggesting that the SlHYL1‐SlRBP1 module is transcriptionally responsive to GA status and may form a positive feedback loop with GA metabolism to regulate plant height. In summary, these results indicate that SlHYL1 and SlRBP1 promote plant height in tomato by additively enhancing the expression of GA biosynthesis genes in an indirect manner.

## DISSCUSION

In this study, we identified SlHYL1 and its interacting partner SlRBP1 as positive regulators of tomato plant height. Mechanistically, this module promotes GA accumulation primarily by upregulating the expression of the GA‐biosynthetic genes *SlCPS* and *SlKAO*, thereby enhancing stem cell elongation and proliferation.

Plant height is determined by both internode number and length. Interfering with *SlHYL1* and *SlRBP1* expression slightly increased internode number but markedly reduced internode length (Figures 1c–e, 3c–e), whereas *SlHYL1‐*overexpression lines showed no opposite phenotype (Figure 1a–e). One possible explanation is that endogenous SlHYL1 levels are sufficient for normal physiological activity in tomato. Moreover, as in *Arabidopsis*, SlHYL1 interacts with the endonuclease SlDCL1 and promotes miRNA biogenesis. However, SlHYL1 overexpression does not enhance the processing process of SlDCL1 (Dong et al., 2008; Fang & Spector, 2007; Wang et al., 2026). Internode length depends on both cell size and cell number. In *SlHYL1‐RNAi* and *SlRBP1‐RNAi* lines, reduced cell length, width, and number were observed in longitudinal sections, contributing to shorter internodes and dwarfism (Figures 1f–i, 3f–i). Cell size and number result from cell expansion and division. RNA‐seq results showed that 13 of 17 expressed expansin genes were downregulated to varying degrees, and qRT‐PCR confirmed this trend, consistent with reduced cell size in *SlHYL1‐RNAi* plants (Figure 4g–j). Cell division and expansion are precisely controlled by a complex regulatory network involving Cyclins, CDKs, and their inhibitors KRPs (Inzé & De Veylder, 2006). The downregulation of these genes in our RNAi lines (Figure 4c–g) is consistent with the observed reduction in both cell size and number, in line with previous reports in tomato (Czerednik et al., 2012; Zhang et al., 2025). Notably, in tomato, SlRBP1 was previously shown to positively regulate the division and expansion of pericarp cells in tomato fruit (Cheng et al., 2025); here, our results extend its roles in stem cell expansion and proliferation.

GAs are crucial in controlling different facets of plant growth, particularly in the determination of plant height, so their levels are subject to strict regulation (Davière & Achard, 2013; Wang et al., 2020). It is noteworthy that two GA precursor synthase genes, *SlCPS* and *SlKAO*, exhibited significant downregulation, which led to a substantial reduction in GA compounds in the *SlHYL1‐RNAi* and *SlRBP1‐RNAi* lines (Figure 5b,f). Evidence suggests that GA precursor synthetic genes are vital for maintaining active GA levels, and their dysregulation can result in decreased plant height and shortened internodes (Regnault et al., 2014). For example, two transposon mutant lines in rice, *oscps1‐1* and *oscps1‐2*, exhibit severe dwarfism and loss of fertility (Sakamoto et al., 2004). In *Arabidopsis*, two *KAO* genes, *KAO1* and *KAO2*, whether in their single or double mutations, both lead to varying degrees of dwarfism (Regnault et al., 2014). Genetically, the SlHYL1‐SlRBP1 double‐interfering lines were shorter than their single parents, with further reduced expression of two GA biosynthesis genes, *SlCPS* and *SlKAO*, supporting the fact that SlHYL1 and SlRBP1 may additively regulate GA biosynthesis, thereby promoting plant height (Figure 6a–c, h). Interestingly, despite the reduced GA content, we observed upregulation of certain GA synthesis genes (*SlGA20ox1* and *SlGA3ox2*) and downregulation of some GA degradation genes (*SlGA2ox1/2/5/6*) (Figure 5f). This seemingly contradictory pattern likely reflects feedback regulation in response to the reduced GA precursor pool, a phenomenon also observed in GA‐deficient *Arabidopsis* mutants (Chiang et al., 1995; Regnault et al., 2014).

HYL1, also known as DRB1, plays a significant role in plant growth and development mediated by miRNA biogenesis (Han et al., 2004). We further investigated the impact of the SlHYL1‐SlRBP1 module on miRNA biogenesis. Surprisingly, unlike SlHYL1, SlRBP1 had minimal effects on miRNA biogenesis (Figure S7j). Furthermore, the transcriptional levels of pri‐miRNAs and miRNA target genes also confirmed the above findings, and no corresponding overall upregulation or downregulation was shown in *SlRBP1‐RNAi* plants (Figure S7d–i). These results indicate that SlRBP1 regulates plant height largely independently of the canonical miRNA pathway. This conclusion is consistent with the emerging view that RBPs often possess multifaced functions. A recent review has highlighted that HYL1's functions extend beyond miRNA biogenesis to encompass transcriptional regulation and protein translation (Gonzalo et al., 2024). In tomato, SlRBP1 has been previously characterized as a regulator of chloroplast function, where it promotes translational efficiency of key chloroplast‐associated transcripts through its interaction with the translation initiation factor SleIF4A2 (Ma et al., 2022). Actually, we did not observe significant leaf yellowing in *SlRBP1‐RNAi* lines (Figure 3a), probably because the residual *SlRBP1* transcript level was sufficient to maintain chloroplast function, consistent with Ma et al.'s findings (Figure S5; Ma et al., 2022). In stems, however, *SlRBP1* expression is lower than in leaves, a similar degree of knockdown may have a greater impact on stems. More recently, SlRBP1 was shown to control fruit size by directly regulating the translation of SlFBA7 and SlGPIMT, which drive pericarp cell division and expansion (Cheng et al., 2025). Here, we extend the role of SlRBP1 to stem cell and surmised that SlRBP1, together with SlHYL1, promotes the expression of GA‐metabolic genes at both the transcriptional and translational levels. However, the precise mechanisms underlying this coordination require further investigation. Moreover, SlRBP1 appears to exert distinct functions across different tissues, reflecting tissue‐specific interacting partners and expression patterns. Identifying the tissue‐specific interacting proteins will be important for understanding how SlRBP1 fulfills distinct functions in different tissues.

In conclusion, our study reveals that the SlHYL1‐SlRBP1 module positively regulates tomato plant height by additively promoting GA biosynthesis, primarily through upregulating *SlCPS* and *SlKAO* expression. Furthermore, different from SlHYL1, the SlRBP1‐mediated plant height regulation is independent of the canonical miRNA biogenesis pathway (Figure 7). This work uncovers a previously unrecognized link between RNA‐binding proteins and GA metabolism, providing new insights into the regulatory network underlying plant architecture in tomato.

**Figure 7:**
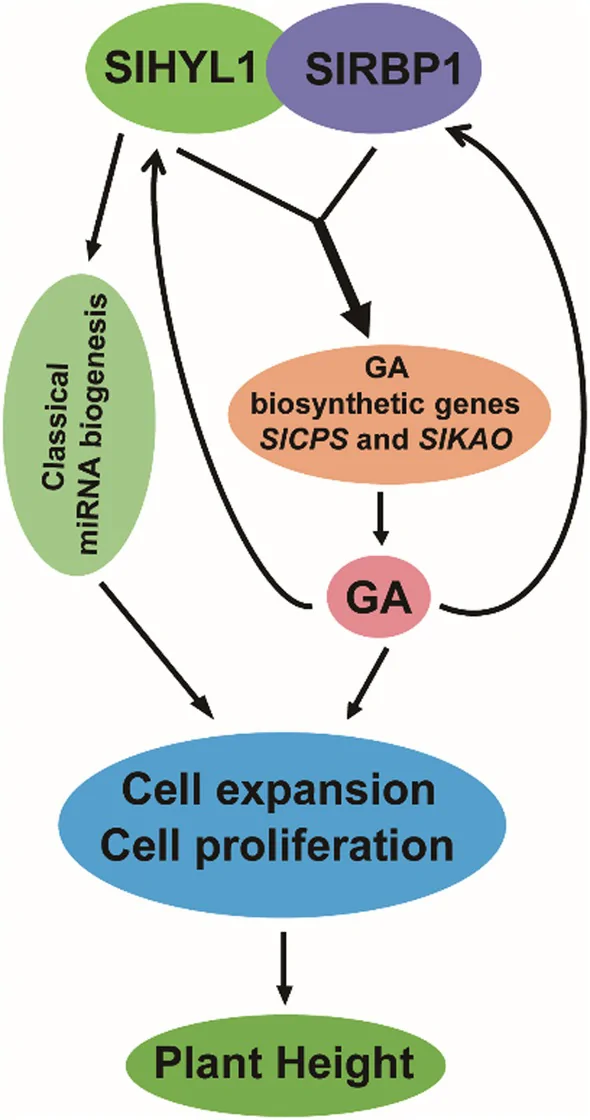
Proposed working model of the SlHYL1‐SlRBP1 module in tomato plant height regulation. SlHYL1 interacts with SlRBP1 to form a functional module that additively enhances the expression of GA‐biosynthetic genes *SlCPS* and *SlKAO*. The resulting GA stimulates cell expansion and proliferation, ultimately increasing plant height. In addition, GA promotes the expression of *SlHYL1* and *SlRBP1*, forming a positive feedback loop. Notably, SlHYL1 is required for classical miRNA biogenesis, whereas SlRBP1 does not affect most miRNAs' biogenesis, indicating SlHYL1 may be involved in miRNA biogenesis‐mediated plant height regulation.

## MATERIALS AND METHODS

### Plant materials, growth conditions, and hormone treatments

The tomato (*Solanum lycopersicum* L.) cultivar “Ailsa Craig” (AC) served as the wild type (WT) in this work, with all transgenic plants derived from this ecotype. All of the plants were grown in the artificial climate chamber at Zhejiang A&F University (Hangzhou, China) at a room temperature of 25 ± 2°C and with a cycle of 14‐h light/10‐h dark. For the organ‐specific expression assay, tissues (shoot apical meristem, roots, young leaves, stems, old leaves, flowers, and fruits) from AC plants were collected, frozen in liquid nitrogen, and stored at −80°C immediately. For phytohormone response analysis, 30‐day‐old AC seedlings were treated with 100 μM GA_3_, IAA, or KT with indicated time. Leaf samples, collected from six plants with three biological replicates, were stored at −80°C after each treatment. In the exogenous GA_3_ spraying experiment, seedlings of 14‐day‐old AC and transgenic seedlings were treated with 100 μM GA_3_ solution every other day for 2 weeks, with each genotype having at least 15 plants.

### Vector construction, plant transformation, generation, and phenotypic analysis

To generate the *SlHYL1‐RNAi* plasmid, the full‐length *SlHYL*1 CDS was PCR‐amplified and cloned into the pBI121‐RNAi vector using ClonExpress II One Step Cloning Kit (Vazyme, China), forming an intermediate plasmid. Subsequently, a *SlHYL1* fragment of approximately 300 bp amplified by PCR was inserted between the *Sac* I and *Sna*B I restriction sites of this plasmid. The *SlRBP1‐RNAi* vector was constructed using the same method. For the 35S::GFP‐*SlHYL1* construct, we fused the *GFP* CDS (without a stop codon) with the full‐length *SlHYL1* CDS and subcloned it into the pBI121 binary vector between the *Bam*H I and *Sac* I sites using the same cloning kit. Primers used for vector construction are listed in Table S3.

The resultant constructs were introduced into *Agrobacterium tumefaciens* strain GV3101, which was then transformed into the tomato cultivar “Ailsa Craig” using plant tissue culture techniques. Homozygous plants of T3 generation were used for phenotypic and molecular characterization.

For phenotypic analysis, at least 15 plants of each genotype were used. Measurements were conducted on the 28th and 42nd days after germination to assess plant height, internode length, and total number of internodes. Plant height is defined as the distance from the cotyledon to the apical growing point. An internode is defined as a stem segment between two adjacent true leaves, with the section from the cotyledon to the first true leaf considered to be internode No. 1, continuing upward.

### RNA extraction and gene expression analysis

According to the product manual, total RNA was extracted using RNAiso Plus reagent (Takara), followed by reverse transcription of RNA using the PrimeScript™ Reverse Transcription Kit (Takara) for mRNAs and the miRNA 1st Strand cDNA Synthesis Kit (Vazyme) for mature miRNAs, respectively. Quantitative reverse transcription PCR (qRT‐PCR) was performed on an Applied Biosystems real‐time PCR machine, utilizing the TB Green® Premix Ex Taq™ kit (Takara). The relative expression levels of each gene were calculated using the 2^–ΔΔCt^ method (Gao et al., 2020), and the *SlACTIN1, NtGAPDH*, and *U6* genes were used as internal controls. Each experiment included three biological replicates. Primers used are listed in Table S3.

### Microscopic observation

Microscopic observation was conducted according to the method of Liu et al. (2023). Briefly, the middle section of the second internode was collected from the stems of 6‐week‐old AC and transgenic plants. Samples were fixed in FAA solution, dehydrated through an ethanol gradient, cleared in xylene, and embedded in Paraplast (Leica, Richmond, VA, USA) for subsequent use. Longitudinal sections of these internodes from the aforementioned materials were prepared, and images were captured using a microscope (Zeiss, LSM510). Cell length and width were measured with ImageJ software. Cell number was determined by dividing the internode length by the average cell length. For each type of plant, 10 internodes were collected, and at least 100 cells were counted in the longitudinal section of each internode, then calculated the average.

### Protein interaction assays

For the yeast two‐hybrid (Y2H) screening, the construction of the tomato cDNA library and the screening of the Y2H library were conducted by Yuanbao Biotechnology Co., Ltd (Nanjing, China). In summary, a mixture of different tissues (root, stem, leaf, flower, and fruit) of AC was sampled for the cDNA library construction. The CDS of *SlHYL1* was inserted into the pGBKT7 vector. After conducting self‐activation and toxicity assessments, this construct was used as bait to screen the interaction protein in the cDNA library. Yeast colonies displaying β‐galactosidase activity on SD/‐Trp‐Leu‐His‐Ade + X‐α‐gal medium were identified as candidate clones. These clones were sequenced to identify the genes of the candidate proteins. For the detail Y2H assay involving SlHYL1 and SlRBP1, both full‐length and truncated CDSs of *SlHYL1* and *SlRBP1* were cloned into pGADT7 (AD) or pGBKT7 (BD) backbone. These AD and BD plasmids were co‐transformed into the yeast strain Y2H Gold according to the experimental design. Positive transformants were identified on SD/‐Trp‐Leu medium. Growth observed on SD/‐Trp‐Leu‐His‐Ade medium indicates interaction between the corresponding proteins.

For the BiFC assay, the full‐length CDSs of *SlHYL1* and *SlRBP1* were fused to the N‐ or C‐terminal fragment of yellow fluorescent protein (YFP) in the pXY103 and pXY105 vectors, respectively. The constructs, *SlHYL1‐nYFP* and *cYFP‐SlRBP1*, were transformed into *A. tumefaciens* strain GV3101 and subsequently co‐infiltrated into *N. benthamiana* leaves. After 36 h of cultivation, YFP fluorescence signals were observed using an Olympus confocal laser scanning microscope to confirm protein interaction.

For the pull‐down assay, the full‐length *SlHYL1* and *SlRBP1* CDSs were cloned into pET28a for 6xHis fusion and pGEX‐4T‐1 for GST fusion by recombination, respectively. These recombinant plasmids were transformed into *E. coli* strain *Transetta* (DE3) (TransGen, CD801‐02), and positive clones were identified. Induction was performed with 0.1 M isopropyl‐β‐D‐thiogalactopyranoside (IPTG) for 16 h at 25°C. The proteins were then purified using GST resin or Ni‐NTA agarose column, following the method described by Zhou, Luo, et al. (2023). Finally, purified samples were denatured in 5XSDS loading buffer and boiled at 95°C for 5 min before immunoblotting. The anti‐HIS (Sangon) and anti‐GST (TransGen) antibodies were used for the immunoblot analysis.

For the Co‐IP assays, the full length of *SlHYL1* and *SlRBP1* CDSs was cloned into pCAMBIA1307‐FLAG and pCAMBIA1300‐MYC plasmids to create the SlHYL1‐FLAG and MYC‐SlRBP1 constructs, respectively. The Co‐IP assay was performed according to Zhao et al. (2025). In brief, these constructs were introduced into *A. tumefaciens* strain GV3101. Two combinations of mixed strains (MYC‐SlRBP1 with either FLAG‐SlHYL1 or FLAG alone) were then infiltrated into the leaves of *N. benthamiana*, followed by incubation in the dark for 24 h, and subsequently exposed to normal light conditions for an additional 48 h. Total protein was extracted from the tobacco leaves using Co‐IP extraction buffer. Protein samples (100 μL) were taken as input, while the remaining was incubated with anti‐FLAG agarose beads (Sigma) for 16 h at 4°C. The immunoprecipitates were washed five times with Co‐IP wash buffer. Proteins were then eluted from the beads using SDS buffer and boiled for 10 mins at 100°C. Both the input samples and the IP samples were examined using immunoblotting with anti‐FLAG (Sigma) or anti‐MYC (Sangon) antibodies. Primers used for all of the vector construction are listed in Table S3.

### RNA‐seq and data analysis

For RNA‐seq analysis, shoot apical meristems were collected from 14‐day‐old AC, *SlHYL1‐RNAi‐17#*, and *SlRBP1‐RNAi‐8#* plants, with three biological replicates for each sample. RNA extraction, library construction, and sequencing were all conducted by Nanjing Yuanbao Biotechnology Co., Ltd. (Nanjing, China). The RNA‐seq data have been submitted to the Genome Sequence Archive of the China National Center for Bioinformation (CNCB), which is under the National Genomics Data Center (NGDC), with the accession number CRA036232. The data can be accessed at: https://ngdc.cncb.ac.cn/. The tomato reference genome and annotation files were obtained from https://ftp.ncbi.nlm.nih.gov/genomes/. The RNA‐seq analysis was conducted with the DESeq2 R package. DEGs were identified using cut‐off values of |Log_2_(Fold Change)| ≥0.585 and a *q*‐value <0.05. t‐SNE analysis and heatmap construction were performed using the Rtsne and pheatmap R packages, separately.

### Quantification of endogenous GAs content

To analyze the various endogenous GA compounds in the AC, *SlHYL1‐RNAi*, and *SlRBP1‐RNAi* lines, the 2nd and 3rd internodes of stems from 6‐week‐old plants were sampled with three independent biological replicates. The endogenous GA content was determined according to Chen et al. (2021) utilizing LC–MS/MS on an Agilent 1290 infinity high‐performance HPLC system coupled with a Sciex‐6500 QTRAP hybrid triple‐quadrupole/linear ion trap mass spectrometer.

### Dual‐luciferase transient expression assay in *Nicotiana benthamiana* leaves

To generate luciferase reporter constructs, 1.5 kb promoter sequences and the full‐length of CDS (without a stop codon) of *SlCPS* and *SlKAO* were amplified, then cloned into pGreenII 0800‐Luc vector to generate pGreenII 0800‐*pSlCPS‐SlCPS and* pGreenII 0800*‐pSlKAO‐SlKAO* by recombination using ClonExpress II One Step Cloning Kit (Vazyme, China). To generate *35S::SlHYL1* and *35S::SlRBP1* effector constructs, the full‐length CDS of *SlHYL1* and *SlRBP1* were PCR‐amplified and cloned into pTA7002 to generate pTA7002‐*SlHYL1* and pTA7002‐*SlRBP1* by recombination. Empty vectors pTA7002 were set as control, respectively. Agrobacterium GV3101, carrying equal concentrations of effector and reporter plasmids (OD_600_ = 0.8), was mixed with 40 μM dexamethasone (DEX) and infiltrated into the leaves of 30‐day‐old *Nicotiana benthamiana*. After the appropriate cultivation period, samples were collected and stored at −80°C. For subsequent analysis methods, refer to Zhang et al. (2021). Primers used for all of the vector construction are listed in Table S3.

## Author Contributions

SG, XZ, and GR designed the research. SG and XZ wrote the paper. XZ, MC, LC, SC, KZ, JH, HZ, MX, and SF conducted the research. LC, BW, and SZ made contributions to growing plant materials. NL, XZ, SG, and YL analyzed the data. GR and SG revised the manuscript. All authors read and approved the manuscript.

## Conflict of Interest

The authors declare no conflicts of interest.

## Supporting information


**Table S1.** Potential candidate proteins interacting with SlHYL1 identified by Y2H library screening.

**Table S2.** List of differentially expressed genes (DEGs), and DEGs co‐regulated by SlHYL1 and SlRBP1 identified from RNA‐seq analysis.

**Table S3.** Primer sequences used in this study.

**Figure S1.** Identification of tomato double‐stranded RNA‐binding protein genes, expression profile, and phylogenetic analysis of SlHYL1.
**Figure S2.** Expression pattern of *SlHYL1* in various tomato tissues and its response to hormone treatments.
**Figure S3.** Characterization of the SlHYL1 knockout mutants.
**Figure S4.** Analysis of transcriptional and protein levels in *SlHYL1* interference and overexpression lines.
**Figure S5.** Transcriptional analysis of *SlRBP1* interference lines.
**Figure S6.** qRT‐PCR analysis of *SlHYL1* and *SlRBP1* expression in reciprocal transgenic lines.
**Figure S7.** Transcriptome and qRT‐PCR analysis of SlHYL1‐ and SlRBP1‐regulated genes, pri‐miRNAs, miRNA target genes, and their corresponding mature miRNAs.
**Figure S8.** Transcriptional analysis of SlHYL1‐specific and *SlRBP1*‐specific regulated genes in AC, double‐RNA interference line, and its single‐knockdown parent.
**Figure S9.** Analysis of the indirect effects of SlHYL1 and SlRBP1 on the expression of GA biosynthesis genes *SlCPS* and *SlKAO*.
**Figure S10.** Transcriptional analysis of *SlHYL1* and *SlRBP1* expression in Moneymaker and *gib3* mutant.

## Data Availability

The raw data of RNA‐seq have been deposited in the Genome Sequence Archive in National Genomics Data Center, China National Center for Bioinformation (https://ngdc.cncb.ac.cn/), under accession number CRA036232. All other data are included in the article and supplementary materials.
