# Supplementary material for: The SlHYL1‐SlRBP1 module integrates gibberellin metabolism to promote plant height in tomato (Solanum lycopersicum)
Source: Plant J. 2026 Nov 1;128(3):e71210. doi: 10.1111/tpj.71210 (PMC13725224; doi:10.1111/tpj.71210)
Supplement: Supplementary file 1 [file TPJ-128-0-s003.docx]

**Supplementary Files**

**Figure S1.** **Identification of tomato double-stranded RNA binding protein genes, expression profile and phylogenetic analysis of SlHYL1.** (a) Expression profile of eight *SlDRBs*. The expression data were obtained from the Tomato Expression Database (http://ted.bti.cornell.edu/). Each square represents the value of log2 (RPKM+1) value. RPKM, Reads Per Kilobase per Million mapped reads. (b) Protein features of HYL1 from *Solanum lycopersicum* and *Arabidopsis thaliana*. The red box indicates the position of the RBD, and the green box indicates the tandem repeat of AtHYL1. Scale bar = 100 aa. (c) Phylogenetic analysis of DRBs from Arabidopsis (AtDRB1/AtHYL1, AT1G09700.1; AtDRB2, AT2G28380.1; AtDRB3, AT3G26932.1; AtDRB4, AT3G62800.1; AtDRB5, AT5G41070.1), tomato (SlDRB1/SlHYL1, Solyc04g076420.2.1; SlDRB2, Solyc11g069460.1.1; SlDRB3, Solyc05g056100.2.1; SlDRB4, Solyc01g056620.2.1; SlDRB5, Solyc03g118950.1.1; SlDRB6, Solyc01g009190.1.1; SlDRB7, Solyc01g056600.2.1 and SlDRB8, Solyc02g091460.2.1), Chinese cabbage (BcpLH, CAB93934.1) and apple (MdDRB1/MdHYL1, AGR54540.1). MEGA 7 program was utilized to construct the phylogenetic tree. (d) Predicted 3D structure of SlHYL1 and AtHYL1 by AlphaFold3. The three-dimensional structure was predicted by AlphaFold3 and visualized in PyMOL (The PyMOL Molecular Graphics System, Schrödinger, LLC). D1, double stranded RNA Binding Domain 1; D2, double stranded RNA Binding Domain 2.

**Figure S2.** **Expression pattern of *SlHYL1* in various tomato tissues and its response to hormone treatments.** (a) Expression level of *SlHYL1* in different tissues of AC were measured using qRT-PCR. RT, root; ST, stem; SAM, shoot apical meristem; AL, adult leaf; JL, juvenile leaf; FL, flower; and FR, fruit. (b) Subcellular localization of SlHYL1 in Nicotiana benthamiana leaves. (c-d) Expression of *SlHYL1* under treatments of 100 μM GA_3_ (C), 100 μM IAA (D), and 100 μM KT. (e) Four-week-old AC seedlings were treated with the hormone solutions respectively, and leaf samples were collected at the indicated time. Each value represents the means ± SD (n = 3). Asterisks indicate statistically signiffcant differences compared to 0 h by t-test (*P<0.05, **P<0.01, ***P<0.001).

**Figure S3. Characterazation of *SlHYL1* CRISPR/Cas9 knockout mutant.** (A) Diagram showing structure with two single RNAs (gRNA1, gRNA2) target three position of *SlHYL1* gene, and three representative genome editing types of *SlHYL1*. Black lines represent the intron, gray boxes represent the exon and the white boxes represent the UTRs. The sequences of guide RNAs, protospacer-adjacent motif are labled blue and green, respectively. In Slhyl mutant, the dashe line ‘-’ colored red indicates a base deletion. (B) Analysis of amino acid sequence deletion of SlHYL1 protein and its three mutants. (C) Phenotypes of Slhyl1 mutants and AC plants at 30 days post sowing. Bar = 10 cm. (D) Plant height of three Slhyl1 mutants and AC plants at 30 days post sowing. Each value represents the means ± SD (n = 6). Asterisks denote that the differences of mutant lines is statistically significant compared to its control AC by t-test (***P<0.001).

**Figure S4.** **Analysis of transcriptional and protein levels in *SlHYL1* interference and overexpression lines.** (a-b) The transcription levels of *SlHYL1* in AC, *SlHYL1*-RNAi and *SlHYL1* overexpression lines. Data are means ± SD, n= 3 biological replicates, Asterisks indicate statistically significant differences compared to AC as determined by t-test (**P < 0.01, ***P < 0.001). (c) Western blot assay showing the protein levels of GFP-SlHYL1 in AC and *SlHYL1* overexpression lines. ACTIN served as the loading control.

**Figure S5.** **Transcriptional analysis of *SlRBP1* interference lines.** Transcription level of *SlRBP1* in AC and *SlRBP1*-*RNAi* lines. Data are mean±SD, with n= 3 biological replicates. Asterisks indicate statistically significant differences compared to AC as determined by t-test (*P < 0.05, **P < 0.01, ***P < 0.001).

**Figure S6. Transcriptional analysis of *SlHYL1* and *SlRBP1* expression in reciprocal transgenic lines.** (a, b) qRT-PCR analysis the expression level of *SlRBP*1 in AC, *SlHYL1*-*RNAi* lines (a) and *SlHYL1* in AC, *SlRBP1*-*RNAi* lines (b). Data are means ± SD, n= 3 biological replicates. Asterisks indicate statistically significant differences between transgenic lines and AC by t-test (ns. no significance).

**Figure S7.** **Transcriptome and qRT-PCR analysis of SlHYL1 and SlRBP1 regulated genes, pri-miRNAs, miRNA target genes and their corresponding mature miRNAs.** (a-b) Volcano plots of the DEGs separately regulated by SlHYL1 (a) and SlRBP1 (b). Red and green dots represent upregulated and downregulated genes, respectively. (c) t-SNE visualization of transcriptomes from different genotypes. (d-e) Boxplot analyses of pri-miRNAs (d) and miRNA targets (e). (f-g) Heatmap of pri-miRNAs and miRNA targets regulated by SlHYL1 and SlRBP1 from RNA-seq data. (h-j) qRT-PCR analyses of pri-miRNAs (h), miRNA targets (i), and mature miRNAs (j) in AC, *SlHYL1*-*RNAi* and *SlRBP1*-*RNAi* lines. Data are mean±SD, with n= 3 biological replicates. Asterisks indicate statistically significant differences compared to AC, as determined by t-test (*P<0.05, **P<0.01, ***P<0.001).

**Figure S8. Transcriptional analysis of *SlHYL1*** **specific and *SlRBP1* specific regulated genes.** qRT-PCR analysis of the expression levels of SlHYL1 specific regulatory genes and SlRBP1 specific regulatory genes in AC, *SlHYL1*-*RNAi*, *SlRBP1*-*RNAi* and double – RNAi lines. Data are means ± SD, with n= 3 biological replicates. Asterisks indicate statistically significant differences compared to AC as determined by t-test (*P < 0.05, **P < 0.01, ***P < 0.001, ns, no significant).

**Figure S9. Analysis of the indirect effects of SlHYL1 and SlRBP1 on the expression of GA biosynthesis genes.** (a) Schematic diagrams of effector and reporter constructs used in the transient dual-luciferase assays. CaMV 35S promoter driving GR-SlHYL1/SlRBP1 was used as effector, and empty vector as a negative control. A 1500 bp promoter sequence of *SlCPS* and *SlKAO* fused with its own full length CDS were used to make the *pSlCPS-SlCPS*:*LUC* and *pSlKAO-SlKAO*:*LUC* reporter, respectively. (b-c) Dynamic expression analysis of *SlHYL1* and *SlRBP1*. qRT-PCR analysis of the expression levels of *SlHYL1* (b) and *SlRBP1* (c) after 30 mM Dexamethasone induced for 0.25, 1, 6, and 24 h, respectively. (d-e) Transient dual-luciferase assay of SlHYL1 and SlRBP1 transactivates the expression of *SlCPS* (d) and *SlKAO* (e) in tobacco leaves. Data are means ± SD, with n= 3 biological replicates. Asterisks indicate statistically significant differences compared to treatment timepoint of 0.25 h as determined by t-test (*P < 0.05, **P < 0.01, ***P < 0.001).

**Figure S10. Transcriptional analysis of *SlHYL1* and *SlRBP1* expression in *gib3*.** qRT-PCR analysis the transcription level of *SlHYL1* and *SlRBP1* in four-week old MM (*Solanum lycopersicum* cv Moneymaker) and *gib3* (GA biosynthetic mutant). Data are means ± SD, with three biological replicates. Asterisks indicate statistically significant differences compared to AC as determined by t-test (*P < 0.05).

**Table S1.** Potential candidate proteins interacting with SlHYL1 identified by Y2H library screening

**Table S2.** List of differentially expressed genes (DEGs) and DEGs co-regulated by SlHYL1 and SlRBP1 identified from RNA-seq analysis

**Table S3.** Primer sequences used in this study
